# Air-Stimulated ATP Release from Keratinocytes Occurs through Connexin Hemichannels

**DOI:** 10.1371/journal.pone.0056744

**Published:** 2013-02-15

**Authors:** Travis P. Barr, Phillip J. Albrecht, Quanzhi Hou, Alexander A. Mongin, Gary R. Strichartz, Frank L. Rice

**Affiliations:** 1 Pain Research Center, Department of Anesthesiology, Perioperative and Pain Medicine, Brigham & Women's Hospital, Boston, Massachusetts, United States of America; 2 Albany Medical College, Center for Neuroscience and Neuropharmacology, Albany, New York, United States of America; 3 Department of Biological Chemistry and Molecular Pharmacology, Harvard Medical School, Boston, Massachusetts, United States of America; University of Arizona, United States of America

## Abstract

Cutaneous ATP release plays an important role in both epidermal stratification and chronic pain, but little is known about ATP release mechanisms in keratinocytes that comprise the epidermis. In this study, we analyzed ATP release from cultured human neonatal keratinocytes briefly exposed to air, a process previously demonstrated to trigger ATP release from these cells. We show that exposing keratinocytes to air by removing media for 15 seconds causes a robust, long-lasting ATP release. This air-stimulated ATP release was increased in calcium differentiated cultures which showed a corresponding increase in connexin 43 mRNA, a major component of keratinocyte hemichannels. The known connexin hemichannel inhibitors 1-octanol and carbenoxolone both significantly reduced air-stimulated ATP release, as did two drugs traditionally used as ABC transporter inhibitors (glibenclamide and verapamil). These same 4 inhibitors also prevented an increase in the uptake of a connexin permeable dye induced by air exposure, confirming that connexin hemichannels are open during air-stimulated ATP release. In contrast, activity of the MDR1 ABC transporter was reduced by air exposure and the drugs that inhibited air-stimulated ATP release had differential effects on this transporter. These results indicate that air exposure elicits non-vesicular release of ATP from keratinocytes through connexin hemichannels and that drugs used to target connexin hemichannels and ABC transporters may cross-inhibit. Connexins represent a novel, peripheral target for the treatment of chronic pain and dermatological disease.

## Introduction

Unlike most cells in the body, keratinocytes lie at the interface with the external environment where they form the outermost layer of the skin, the epidermis. The epidermis is a dynamic, stratified structure formed by continually proliferating and differentiating keratinocytes that surround the sensory nerve endings of several subtypes of C- and Aδ-fibers [1]. These fibers play an important role in tactile sensation and nociception and express numerous ligand-gated receptors that can regulate their signaling [2], [3]. Keratinocytes have been implicated in mechano- and thermosensation, as well as peripheral pain mechanisms due to their release of molecules that activate such receptors, including β-endorphin, calcitonin gene-related peptide (CGRP) and ATP [4], [5], [6].

Cutaneous ATP release is an important signal for epidermal homeostasis as well as the generation of acute and chronic pain. Signaling among keratinocytes through the release of ATP influences their proliferation and differentiation, thereby playing a major role in the creation of the stratified structure of the epidermis and maintaining epidermal homeostasis [7]. During acute tissue injury, such as cuts and abrasions, excessive ATP release from damaged keratinocytes causes pain by activating excitatory purinergic receptors on nociceptive sensory nerve endings [8], [9], [10]. Lower levels of ATP released by keratinocytes during epidermal homeostasis and in response to mild mechanical and thermal stimulation may participate in normal tactile sensation and also contribute to the spontaneous pain and tactile hypersensitivity that occurs under chronic pain conditions, when nociceptive nerve endings become sensitized [11], [12]. Release of ATP from keratinocytes may also be increased during chronic pain [5]. Consistent with a contribution of epidermal ATP release to chronic pain, cutaneous administration of purinergic receptor antagonists reduces nociceptive behavior in a variety of animal models of chronic pain [13], [14], [15], [16].

Despite the importance of ATP in epidermal homeostasis, tactile sensation and nociception, little is known about the mechanisms of keratinocyte ATP release. Mechanical and thermal stimulation, low pH and hypo-osmotic stimulation have all been shown to result in ATP release from keratinocytes, but the relevant mechanisms were not identified [11], [12], [17], [18]. Recently, we showed that activation of keratinocyte voltage-gated sodium channels triggers ATP release and that this mechanism appears to be up-regulated under chronic pain conditions [5]. These results may indirectly suggest vesicular release, although such a mechanism has never been demonstrated in keratinocytes. Several non-vesicular ATP release mechanisms have been proposed, but many remain controversial and are complicated by the non-specificity of available inhibitors [19], [20].

Air exposure has also been shown to cause ATP release from cultured keratinocytes, though this release mechanism was not previously investigated [21]. Keratinocyte interactions with air may be an important signal to trigger epidermal stratification, as cultured keratinocytes will not form a fully stratified epidermis unless they are brought to the air interface [22], [23]. Given the importance of keratinocyte ATP release in epidermal stratification and nociception, combined with the lack of information about keratinocyte ATP release mechanisms, the goal of the present study was to characterize air-stimulated ATP release by analyzing its time course, the extent of intracellular ATP depletion and the mechanism involved.

## Materials and Methods

### Cell Culture

Neonatal normal human epidermal keratinocytes (NHEK, Lonza, Basel, Switzerland) were cultured in chemically defined keratinocyte growth media (KGM-CD, Lonza) supplemented with 0.5% penicillin/streptomycin (Invitrogen, Carlsbad, CA) and maintained at 37°C and in an atmosphere of 95% air/5% CO_2_. NHEK were plated in collagen coated 35 mm dishes at a cell density between 3,500 and 10,500 cells/cm^2^ and cultured until they were 70–90% confluent (proliferating cultures), 100% confluent (confluent cultures), or cultured until they reached 100% confluency and then further cultured in KGM-CD containing 2 mM calcium for 5 days (differentiated cultures). Cultures were fed every 2–3 days by completely aspirating the media and replacing it with fresh KGM-CD.

### Quantitative RT-PCR

RNA was isolated using the Trizol method from 3 proliferating and 3 differentiated cultures that were plated simultaneously. Quality and quantity of isolated RNA were analyzed using a Nanodrop spectrophotometer (Thermo Fisher Scientific, Waltham, MA). RNA was converted to cDNA using an iScript kit (Biorad, Hercules, CA) with 2 µG total RNA in 20 µL reactions with an Ependorf Mastercycler (Hamburg, Germany) using the following protocol: 5 minutes at 25°C, 30 minutes at 42°C and 5 minutes at 85°C. Expression of genes of interest was determined by qPCR using iTaQ fast SYBR green master mix (Biorad) in 20 µL reactions. Amplification was conducted using gene-specific primers for targets of interest that were synthesized by Invitrogen based on published sequences, validated primers from Qiagen (Valencia, CA), or provided by Dr. Spiro Getsios (Northwestern University). A complete list of the primers used are listed in Table 1. All primers were used at 450 nM and the same primer/SYBR green mix was used to amplify all samples. qPCR reactions were conducted in duplicates using the following protocol: a single 3 minute step at 95°C followed by 40 cycles of 10 seconds at 95°C, 10 seconds at 55°C, and 30 seconds at 72°C. A Biorad CFX96 qPCR system was used with a C1000 thermocycler and data were analyzed with CFX Manager V1.5 (Biorad). Target gene expression was normalized to the housekeeping gene YWHAZ, but similar results were also found when normalized to GAPDH (data not shown). All qPCR targets produced single peak melt curves, except for CFTR. This may be due to splice variants, but since CFTR has been previously shown in keratinocytes and was not the protein identified in pharmacological experiments, the multiple products were not investigated further.

**Table 1:** Primers used for qPCR analyses.

Target	Sequence (5′→3′)	Source
YWHAZ, forward	GCCACAATGTTCTTGGCCCATCAT	Dr. Spiro Getsios, Northwester University
YWHAZ, reverse	TGGTTGGTGACAAGACAGAAGGCT	Dr. Spiro Getsios, Northwester University
Keratin 14	Proprietary, Validated Primer	Qiagen
Keratin 10, forward	AAACCGCAAAGATGCTGAAGCCTG	Dr. Spiro Getsios, Northwester University
Keratin 10 reverse	TCAAGGCCAGTTGGGACTGTAGTT	Dr. Spiro Getsios, Northwester University
Loricrin, forward	TCTCATGATGCTACCCGAGGTTTG	Dr. Spiro Getsios, Northwester University
Loricrin, reverse	GGGTTGGGAGGTAGTTGTACAGAA	Dr. Spiro Getsios, Northwester University
Involucrin, forward	TGCCTGAGCAAGAATGTGAG	Invitrogen
Involucrin, reverse	AGCTGCTGATCCCTTTGTGT	Invitrogen
Connexin 43	Proprietary, Validated Primer	Qiagen
CFTR	Proprietary, Validated Primer	Qiagen
Pannexin 1	Proprietary, Validated Primer	Qiagen
Pannexin 2	Proprietary, Validated Primer	Qiagen
P2X7, forward	CTTTCTCAAAACAGAAGGCCAAGA	Invitrogen
P2X7, reverse	CAACCTCGGTCAGAGGAACAGA	Invitrogen
MDR1, forward	CACCCGACTTACAGATGATG	Invitrogen
MDR1, reverse	GTTGCCATTGACTGAAAGAA	Invitrogen
MRP1, forward	GCCGAAGGAGAGATCATC	Invitrogen
MRP1, reverse	AACCCGAAAACAAAACAGG	Invitrogen

### Air-stimulated ATP Release Assays

#### Time course experiments

Dishes were rinsed twice with 2 mL of HEPES buffered “basal media” containing (in mM): 135 NaCl, 3.8 KCl, 1.2 MgSO_4_, 1.3 CaCl_2_, 1.2 KH_2_PO_4_, 10 HEPES and 10 D-glucose, pH 7.4. Rinses were done quickly to minimize air exposure (1–2 seconds). Cells were then pre-incubated at room temperature for one hour to allow degradation of ATP released during rinses. Immediately before air exposure, a 100 µL baseline sample was taken. Cells were then exposed to air by aspirating all of the media and slowly (over 1–2 seconds) pipetting 2 mL of basal media onto the side of the dish after 15 seconds had passed. For controls, only half of the media was exchanged to prevent air exposure and provide a control for potential ATP release from mechanical stimulation. Samples (100 µL) of extracellular media were taken at each time point and immediately placed into wells of a white 96-well plate on ice containing 50 µL mammalian cell lysis buffer solution (ATPlite kit, Perkin Elmer, Waltham, MA) to stabilize ATP. Data were analyzed as changes from baseline release, because an increase in protein expression in differentiated cells prevented normalization to total protein (data not shown).

#### Intracellular ATP levels after air exposure

Experiments were conducted as described for the time course experiments. At 7 and 60 minute time points (times near peak release and return to baseline), cells were lysed by replacing media with 500 µL of mammalian cell lysis buffer solution. Samples (100 µL) of the lysates were diluted 10-fold in basal media to fit within the calibration range of the ATP assay. ATP content in all samples was calculated as % control at the 7 minute time point.

#### Pharmacological analysis of air-stimulated ATP release

Cells were treated the same as in time course experiments, except on the second rinse with basal media, only 1 mL was added. Ten minutes before the one hour pre-incubation period was complete, 1 mL of the appropriate inhibitor was added at double the final desired concentration. The following inhibitors of non-vesicular ATP release pathways were purchased from Sigma Aldrich (St. Louis, MO) and used at the final concentrations indicated in parentheses: the connexin hemichannel blocker 1-octanol (2 mM), the dual connexin/pannexin hemichannel blocker carbenoxolone (100 µM), the P2X7 channel blocker brilliant blue G (BBG, 1 µM), the anion channel blockers phloretin (100 µM), 5-nitro-2-(3-phenylpropylamino)-benzoate (NPPB, 100 µM) and gadolinium chloride (50 µM), the CFTR transporter inhibitor glibenclamide (200 µM), the MDR1 transporter inhibitor verapamil (10 µM) and the MRP1 transporter inhibitor probenecid (1 mM) [24], [25], [26], [27], [28], [29], [30], [31], [32]. Phloretin, NPPB, glibenclamide and probenecid were dissolved in DMSO so that the final concentration of DMSO in the test media was ≤0.1%. All other agents used were soluble in basal media. Air exposure was conducted as described for time course experiments, except basal media was now added with or without (vehicle) the appropriate inhibitor. Samples (100 µL) of extracellular media were collected immediately before (baseline) and 7 minutes after air exposure, near the time of peak ATP release.

### Quantification of ATP Levels

ATP concentrations were determined using a luciferin/luciferase assay kit (ATPlite, PerkinElmer). This kit has a long reaction half-life and was validated using standard curves of ATP to ensure measurements were stable throughout the time course of experimentation and plate reading. Luminescence was measured using a Victor3 1420 plate reader (PerkinElmer) with a 535 nm emission filter for 2 seconds. Luminescence was measured 3 times for each well and then averaged for a single data point. For each experiment, values were plotted on the line of best fit for a 6-point logarithmic standard curve (0.01 µM–3.2 µM ATP) using Origin 6.0 (OriginLab, Northampton, MA).

### Cell Viability Assay

Confluent cultures were put through the same procedure as described for the pharmacological analysis experiments. Sixty minutes after air exposure, cells were rinsed twice quickly with basal media, fixed for 20 minutes with 4% paraformaldehyde, and labeled with propidium iodide (PI, 5 µL/mL) for 90 minutes. Cells were then rinsed three times with PBS for 5 minutes per rinse. Five images were captured of both the light field and PI fluorescence for each dish. Image capture settings were identical for all images and set so that all nuclei considered positive for PI were saturated (i.e. appeared as easily identifiable solid white dots). Light field and PI images were merged and then PI positive and total cells were manually tagged and counted using Neurolucida (MBF Biosciences, Williston, VT).

### Assessment of Connexin Hemichannel Activity

Uptake of a connexin hemichannel permeable dye (lucifer yellow, LY) was measured to assess the extent of hemichannel opening in response to air exposure alone and in the presence of ATP release inhibitors. Confluent cultures were treated the same as described for the pharmacological analysis experiments, with 10 minute pre-incubation of inhibitors. After media was removed for 15 seconds, it was replaced with 1 mL of test solution including 500 µM LY (Sigma Aldrich, St. Louis, MI) and the appropriate inhibitor. Sixty minutes after the addition of dye, cells were quickly rinsed twice with basal media containing 2 mM 1-octanol to remove residual LY while minimizing leakage of absorbed dye. LY fluorescence was read on a Victor3 1420 plate reader for 1 second per measure using 405 nm excitation and 535 nm emission filters. Three fluorescence measures were averaged for each data point.

### Assessment of MDR1 Transporter Activity

Activity of the MDR1 ABC transporter was determined by measuring the uptake of a radiolabeled substrate of this transporter, [^3^H]-digoxin. [^3^H]-digoxin is a membrane-permeable molecule that is excluded from the cell based on active transport by MDR1. Proliferating cultures were treated similarly to pharmacological analysis experiments. Ten minutes before the end of pre-incubation, 500 µL of the 1 mL of remaining media was removed and replaced with 500 µL of media with the appropriate inhibitor at twice the desired concentration or basal media alone. After 10 minutes, 500 µL was again removed and replaced with [^3^H]-digoxin-containing media (50 nM final concentration with specific activity of 20 Ci/mmol, American Radiolabled Chemicals, St. Louis, MO) with or without the appropriate inhibitor, both at twice the desired concentration. For the air exposure group, all media was removed for 15 seconds and replaced with 1 mL of [^3^H]-digoxin-containing basal media. After 5 minutes, cells were lysed with 2% SDS/8 mM EDTA and 500 µL samples were counted in 5 mL of Ecolite(+)™ Liquid Scintillation Cocktail (MP Biomedicals, Solon, OH) for 5 minutes on a Beckman Model LS8500 (Beckman, Inc; Palo Alto, CA) scintillation counter.

### Statistical Analysis

Data were analyzed using Statistica 6.0 (Statsoft). qPCR and intracellular ATP data were analyzed using independent sample *t*-tests. General linear model two-way ANOVA were used to analyze time course and pharmacological analysis data, with subsequent one-way ANOVA and Dunnette's post-hoc tests. PI, LY and [^3^H]-digoxin data were analyzed using one-way ANOVA with Dunnette's post-hoc tests. Differences were considered significant if *p*<0.05.

## Results and Discussion

### Keratinocytes Express Multiple ATP Release Pathways

**Figure 1:**
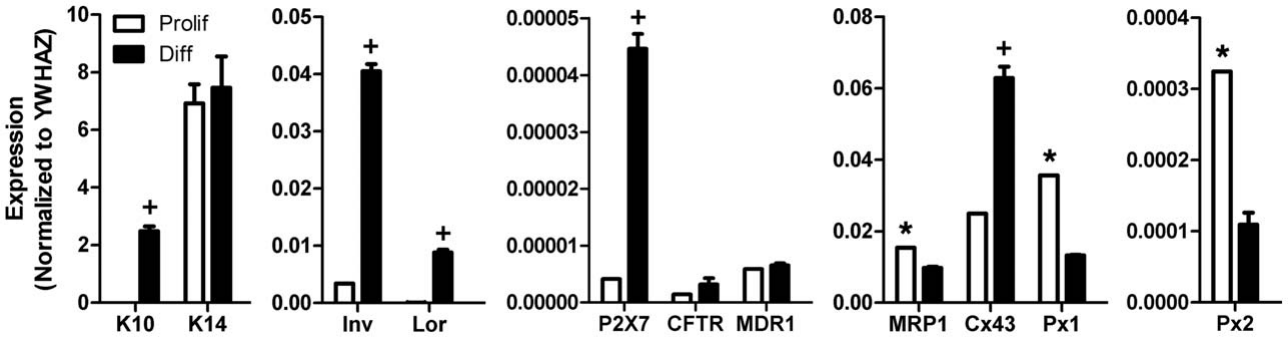
mRNA expression of putative ATP release pathways in NHEK. qPCR was used to assess potential ATP release pathways present in proliferating (white bars) and calcium differentiated (black bars) NHEK cultures. All non-vesicular ATP release pathways investigated were present in NHEK cultures. The P2X7 purinergic receptor, which is proposed to form an ATP permeable pore through an interaction with pannexins, was significantly increased in differentiated cells. The ABC transporters CFTR and MDR1 were not altered by differentiation, although the MRP1 ABC transporter showed a small, but significant increase. The hemichannel component connexin 43 (Cx43) was also increased by differentiation, while the other hemichannel components pannexin 1 and 2 (Px1 and 2) were significantly decreased. Large increases in the differentiation markers keratin 10 (K10), loricrin (Lor) and involucrin (Inv) verified the differentiation status of calcium treated cultures. The basal keratinocyte marker keratin 14 (K14) was similarly present in both proliferating and differentiated cultures. Values are shown relative to the housekeeping gene YWHAZ and represent data from 3 samples +/− SEM. Due to differences in expression levels, multiple Y axes are used. *significantly decreased in differentiated cells, ^+^significantly increased in differentiated cells (*p*<0.05).

The presence of potential non-vesicular ATP release pathways in NHEK cultures was determined by qPCR analyses. Transcripts for all investigated ATP release pathways were detected in both proliferating and calcium differentiated cultures (Figure 1). Calcium treated cultures showed significantly higher levels of keratin 10, involucrin and loricrin (*p*<0.0001), confirming increased keratinocyte differentiation. Levels of P2X7 and connexin 43 were also significantly higher following calcium differentiation (*p*<0.001), in agreement with previous studies [7], [33]. MRP1 showed a small, but significant decrease in differentiated keratinocytes (*p*<0.01), while the other ABC transporters MDR1 and CFTR were relatively unchanged (*p* = 0.3124 and 0.1957, respectively). A previous study reported increased levels of MRP1 and MDR1 using a longer time period of calcium differentiation [34]. In contrast to evidence from immunostaining studies, mRNA levels for pannexin 1 and pannexin 2 were significantly higher in proliferating keratinocytes (*p*<0.0001 and 0.01, respectively) [35]. Such differences in the localization and measure of mRNA and protein signals may result from non-specificity of antibodies or translational and post-translational protein regulation. We have previously shown that voltage-gated sodium channels have a wider distribution of mRNA compared to immunostaining in keratinocytes [5].

### Characteristics of Air-stimulated ATP Release

**Figure 2:**
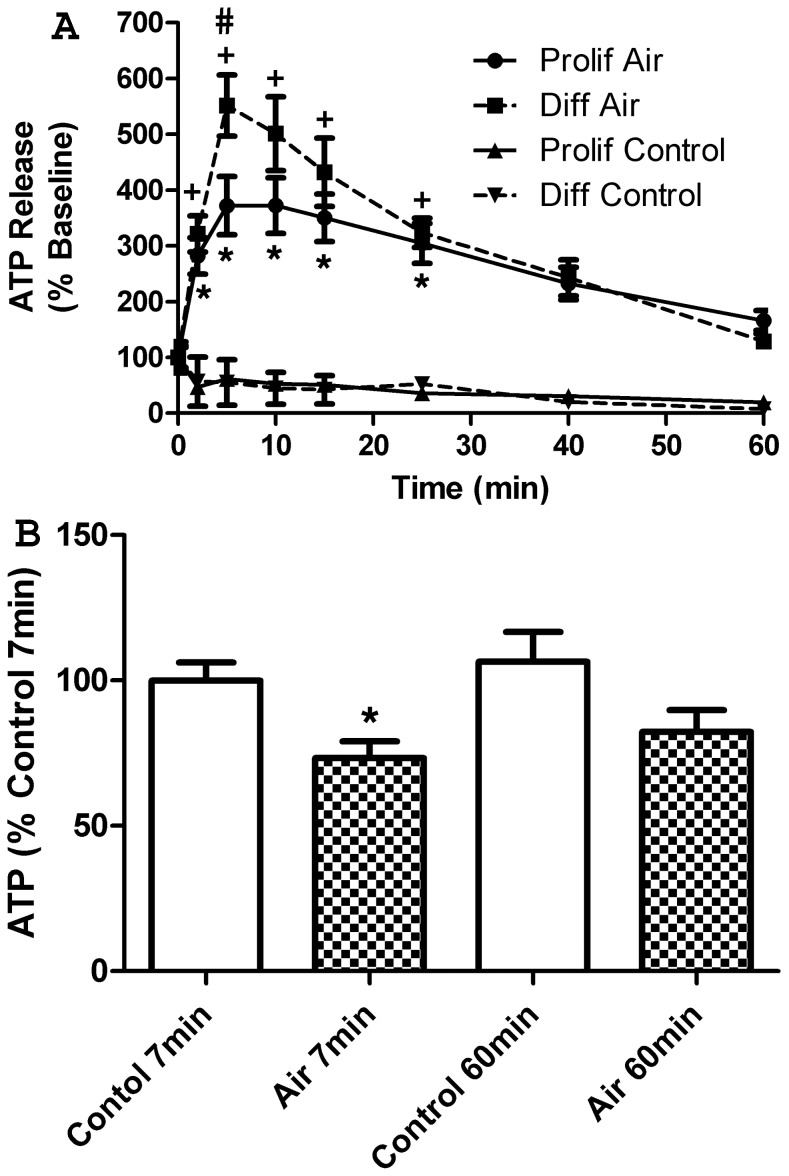
Time course and extent of intracellular ATP release. A. Time course of air-stimulated ATP release. Extracellular ATP levels were quantified following a 15 second air exposure (Air) or an exchange of half the media (Control) in proliferating (Prolif) and calcium differentiated (Diff) NHEK cultures. Air-stimulated ATP release was significantly higher in differentiated cultures at the five minute time point, but remained significantly elevated for 25 minutes in both cultures. Values represent the mean +/− SEM for 13 air-exposed and 2 control dishes per group shown as % baseline. * *p*<0.05 vs. 0 minute Prolif, + *p*<0.05 vs. 0 minute Diff, # *p*<0.05 Diff vs. Prolif. **B. Intracellular ATP following air exposure.** To determine the extent of intracellular ATP reduction after air exposure, levels of ATP in cell lysates of air-exposed and control confluent NHEK cultures were measured. Air-exposed cultures showed a significant reduction in intracellular ATP at the time of peak ATP release (7 minutes after exposure). This reduction was no longer significant by 60 minutes after air exposure. Values represent averages +/− SEM of 6 dishes per group, * *p*<0.05 vs. control 7 minutes.

**Figure 3:**
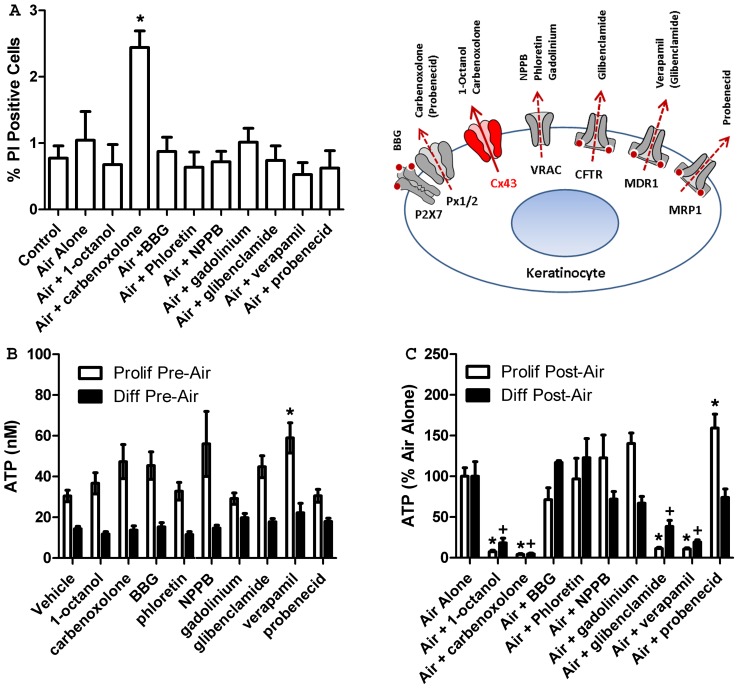
Pharmacological analysis of the air-stimulated ATP release mechanism. A. propidium iodide (PI) labeling. PI labeling was measured to ensure that air exposure and drugs used in inhibition experiments did not compromise cell membranes. Only pretreatment with carbenoxolone significantly increased PI labeling and this remained below 3% of cells. All values represent averages +/− SEM of 6 dishes per group, * *p*<0.05 vs. control. **To determine the mechanism of air-stimulated ATP release**, keratinocytes were pre-incubated for 10 minutes with known inhibitors of ATP release pathways before air exposure. ATP levels in the media were assayed just prior to air exposure to determine any effects of the drugs themselves on baseline ATP release (**B**, pre-air) and 7 minutes after air exposure (**C**, post-air) to measure the ability of drugs to inhibit air-stimulated ATP release. **B**. Pre-air ATP levels were significantly lower in differentiated cultures, but similar for most drug treatment groups. **C**. In both proliferating and differentiated cultures, pre-incubation with the connexin hemichannel blockers 1-octanol and carbenoxolone largely abolished air-stimulated ATP release. Glibenclamide and verapamil, drugs traditionally used as ABC transporter blockers, also significantly inhibited air-stimulated ATP release. Post-air ATP levels are expressed as percent of air alone. Values represent 12 (drug treatment) or 24 (air alone) dishes. Error bars are +/− SEM, *,^+^ *p*<0.05 vs. vehicle or air alone for proliferating and differentiated cells, respectively. **Inset. Schematic showing potential keratinocyte ATP release pathways and the major targets of inhibitors used.** Secondary targets discussed in the text are also shown in parentheses. Red dots and arrows represent regulatory ATP binding sites and routes of release, respectively. **Release Pathways.** P2X7: P2X7 ATP receptors, Px1/2: pannexins-1 & 2, Cx43: connexin-43, VRAC: volume-regulated anion channels (unknown molecular identity) and the ATP binding cassette (ABC) transporters CFTR, MDR1 and MRP1.

Exposing keratinocytes to air for 15 seconds caused a robust, long-lasting ATP release that was significantly higher in differentiated cultures (Figure 2a, ANOVA *p*<0.01). This release was not due to cell lysis, as the same procedure did not increase PI staining (Figure 3a, *p* = 0.99). This release was also not merely due to mechanical stimulation, as an exchange of half the media in controls did not result in any detectable ATP release. ATP levels in the media were significantly higher than baseline in both cultures at 2, 5, 10, 15 and 25 minutes (Figure 2a, *p*<0.01), with differentiated keratinocytes showing higher ATP levels at five minutes (*p*<0.05). Given the usually rapid metabolism of extracellular ATP, it seems likely that some release continues throughout this time course, although there may also be a contribution of re-phosphorylated AMP and ADP [36], [37].

The amount of intracellular ATP remaining in air-exposed cells suggests that the ATP released during this process is a sizable proportion of intracellular ATP, although potential changes in synthesis and metabolism were not analyzed. Seven minutes after treatment, air-exposed cells had a significant reduction (∼25%) in total intracellular ATP (Figure 2b, *p*<0.01). Intracellular ATP levels in air-exposed cultures recovered slightly by 60 minutes and were no longer significantly different than control (*p* = 0.089). In standard keratinocyte cell culture procedures, feeding cells requires replacement of media every 2–3 days, which routinely involves brief air exposure. The fact that such air-exposed cells maintain viable cultures demonstrates that these cells recover from this stimulus and the lack of increased PI staining shows cell membranes are not compromised (Figure 3a).

### Air Exposure Triggers ATP Release Through Connexin Hemichannels

PI labeling confirmed that drugs used to inhibit ATP release pathways cause little, if any harm to the cells (Figure 3a). Only carbenoxolone caused a significant increase in the percentage of PI labeled cells (*p*<0.05) and this still remained less than 3% of total cells. Differentiated keratinocytes had significantly lower baseline extracellular ATP levels compared to proliferating cells (Figure 3b, ANOVA *p*<0.05). This could be due to a change in un-stimulated ATP release or metabolism caused by the calcium differentiation, or simply a consequence of the higher cell density that occurs in differentiated cultures. No inhibitor significantly reduced un-stimulated ATP release, although verapamil resulted in a significant increase (*p*<0.05).

In both proliferating and differentiated keratinocytes, air-stimulated ATP release was almost completely prevented by the connexin hemichannel inhibitors 1-octanol and carbenoxolone (Figure 3c). Carbenoxolone can block both connexins and pannexins, but no inhibitory effect was seen with probenecid, which can also inhibit pannexins [38]. Glibenclamide and verapamil, drugs traditionally used to inhibit ABC transporters, also significantly inhibited air-stimulated ATP release from NHEK cultures (Figure 3c, *p*<0.0001–0.05). The inhibition by both glibenclamide and verapamil suggested the involvement of the MDR1 ABC transporter, since the CFTR ABC transporter is only inhibited by glibenclamide [39].

**Figure 4:**
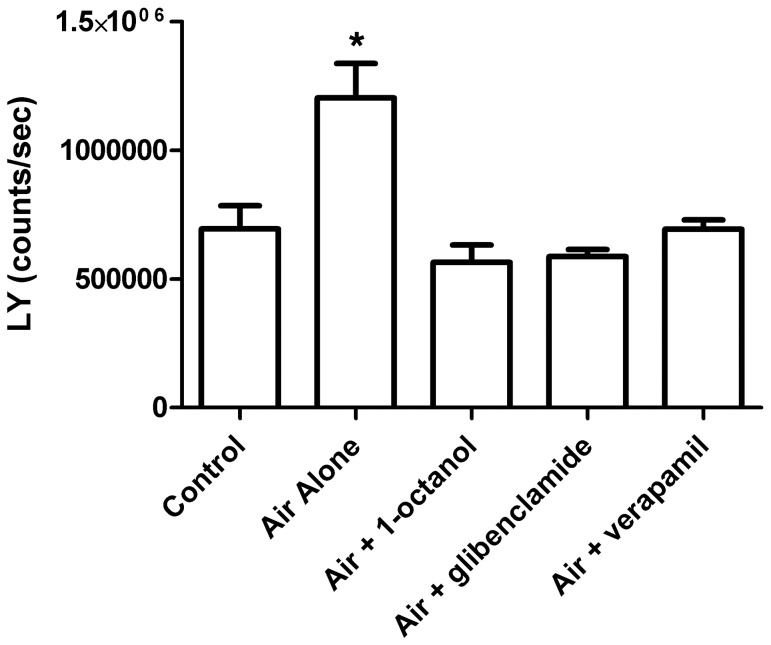
Assessment of connexin hemichannel involvement in air-stimulated ATP release. Uptake of the connexin permeable dye lucifer yellow (LY) was measured to confirm that connexin hemichannels open in response to air exposure and are blocked by the same drugs that prevent ATP release. Cells were pre-incubated with ATP release inhibitors using the same procedure as for the pharmacological analysis assays. LY (500 µM final concentration) was added in the media after air exposure or a change of half the media (control). Sixty minutes later, cultures were rinsed twice with media containing 1-octanol (2 mM) to remove residual LY, but minimize leakage of absorbed LY. LY levels in the cell lysates were then measured. Air exposure significantly increased LY uptake compared to control and the same drugs that inhibited air-stimulated ATP release prevented this increase in LY uptake. This indicates that connexin hemichannels are open during air-stimulated ATP release. Values represent 8 or 9 wells per group +/− SEM, * *p*<0.05 vs. control.

**Figure 5:**
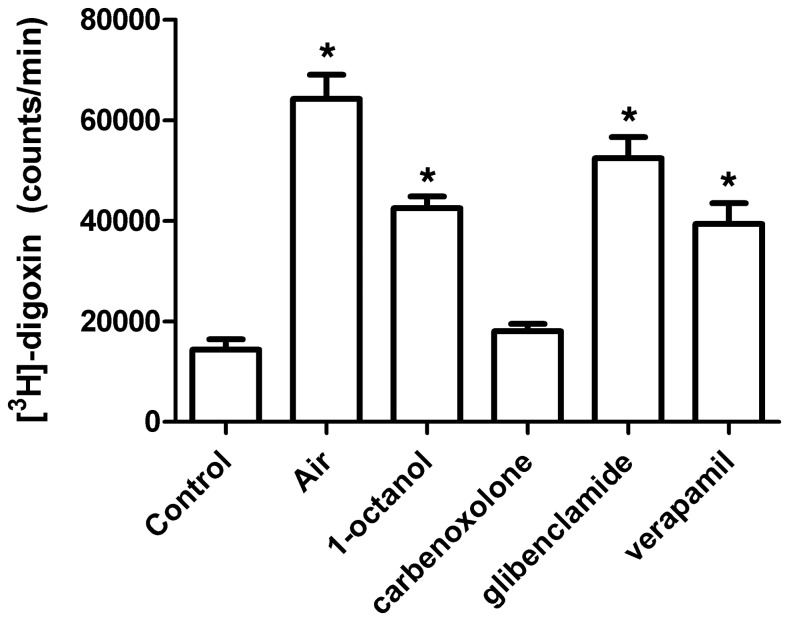
Assessment of MDR1 transporter involvement in air-stimulated ATP release. Exclusion of a radiolabeled substrate of the MDR1 ABC transporter was measured to determine whether activity of this transporter is increased during air-stimulated ATP release. Note that this transporter acts to exclude substrates, so an increase in [^3^H]-digoxin uptake reflects an inhibition of transporter activity. Confluent NHEK cultures were pre-incubated with ATP release inhibitors using the same protocol as for the pharmacological analysis assays. [^3^H]-digoxin (50 nM final concentration) was added in the media after air exposure or a change of half the media (control). After 7 minutes, the time of peak air-stimulated ATP release, [^3^H]-digoxin levels in cell lysates were measured. Air exposure significantly increased [^3^H]-digoxin uptake, as did the ABC transporter inhibitors glibenclamide and verapamil. The connexin blocker 1-octanol also significantly inhibited uptake, while the other connexin blocker carbenoxolone was without effect. The inhibition of MDR1 following air exposure, combined with the disparate effects of ATP release inhibitors, indicates that this transporter is not involved in air-stimulated ATP release. Values represent 6 wells per group +/− SEM, * *p*<0.05 vs. control.

An interaction between the CFTR ABC transporter and connexins has recently been suggested in airway epithelial cells [40]. To determine whether both the MDR1 ABC transporter and connexin hemichannels were involved in air-stimulated ATP release, uptake of the connexin permeable dye LY and exclusion of the radiolabeled MDR1 substrate [^3^H]-digoxin were measured following air exposure (Figures 4 and 5, respectively). Air exposure significantly increased the uptake of LY (Figure 4, *p*<0.001), consistent with an opening of connexin hemichannels in response to this stimulus. This increase in LY uptake was blocked by the connexin hemichannel blocker 1-octanol, but also by the ABC transporter inhibitors glibenclamide and verapamil (*p* = 0.7814–1.0). The increase in LY uptake following air exposure, but not in the presence of drugs that inhibit air-stimulated ATP release, indicates that connexin hemichannels are open during air-stimulated ATP release.

In contrast, [^3^H]-digoxin exclusion experiments showed that activity of MDR1 is inhibited by air exposure, thus it is unlikely that this transporter is involved in air-stimulated ATP release (Figure 5). Air exposure significantly increased intracellular levels of [^3^H]-digoxin (*p*<0.0001), indicating a reduced capacity of MDR1 to transport this substrate out of the cell. A similar effect was seen for the known MDR1 inhibitors glibenclamide and verapamil in the absence of air exposure (*p*<0.0001). Unexpectedly, 1-octanol also significantly increased uptake of [^3^H]-digoxin (*p*<0.0001), suggesting this drug may also inhibit MDR1. Carbenoxolone was without effect (*p* = 0.9040), demonstrating that not all drugs that block air-stimulated ATP release also inhibit MDR1. It seems possible that the reduction in intracellular ATP, required for MDR1 transport, could be responsible for the inhibition of this transporter following air exposure.

Taken together, our data provide strong evidence that air exposure stimulates ATP release from keratinocytes through connexin hemichannels and that the inhibitory effects of the ABC transporter inhibitors glibenclamide and verapamil on this release may be due to non-specific actions on these hemichannels. It is also possible that the inhibitory effects of glibenclamide and verapamil on connexin hemichannels are through an intermediate target. For example, these drugs are known to inhibit ion channels expressed by keratinocytes, which may in turn have an influence on connexin hemichannels [41], [42], [43], [44].

Previous studies have provided compelling evidence that, at least in some cell types, ATP release can occur through connexin hemichannels [45]. Expression of connexin 43, the major connexin in keratinocytes, is increased in suprabasal keratinocytes *in vivo* [45], [46]. Consistent with these observations, our data showed increased levels of connexin 43 mRNA and a corresponding increase in air-stimulated ATP release in differentiated cultures. While connexin 43 is the most highly expressed connexin in keratinocytes, many other subtypes are present and may also contribute to air-stimulated ATP release. More detailed analyses beyond the scope of this study, possibly utilizing siRNA and connexin inhibiting peptides, would be necessary to determine which specific connexin subtypes are involved.

Connexin hemichannels could also be involved in keratinocyte ATP release in response to other tactile stimuli. For example, connexin hemichannels have been shown to be mechanosensitive and connexin inhibitors reduce calcium waves mediated by ATP release in response to mechanical stimulation of cultured keratinocytes [12], [47]. While it was concluded that the reduced calcium wave propagation in the presence of these drugs was due to blockade of gap junctions between neighboring keratinocytes, a blockade of ATP-permeable hemichannels that opened in response to mechanical stimulation was not ruled out as a contributing factor.

A significant increase in air-stimulated ATP release was seen in the presence of probenecid in proliferating cultures, although the reason for this is unclear (Figure 3c, *p*<0.05). Probenecid did not cause an increase in un-stimulated ATP release, indicating it does not cause ATP release itself (Figure 3b).

### Potential Role for Air-stimulated ATP Release in Epidermal Homeostasis, Barrier Formation and Dermatological Disease

ATP released from keratinocytes through interactions with air may be an important signal in regulating epidermal homeostasis. ATP plays a complex role in epidermal homeostasis and barrier formation by signaling through P2Y and P2X receptors which are primarily restricted to basal and suprabasal keratinocytes, respectively [7]. This is further complicated by the different affinities of purinergic receptors for ATP metabolites. P2Y1 and P2Y2 activation increases DNA synthesis and cell number in submerged keratinocyte cultures and also increases epidermal thickness of skin *in vivo*. In contrast, P2X5 and P2X7 activation reduces keratinocyte number in culture and reduces epidermal thickness *in vivo*. Cutaneous injection of ATP causes epidermal thickening, indicating P2Y receptor-stimulated proliferation predominates over the anti-proliferative effects of P2X receptors. Along with their apparent anti-proliferative effects, P2X receptors also inhibit terminal differentiation and barrier formation [48], [49].

There is evidence that air-stimulated ATP release regulates epidermal homeostasis *in vivo*. Disruption of the epidermal barrier by tape stripping or acetone, a process that would increase air exposure of keratinocytes, results in increased cutaneous ATP release and signaling. Tape stripping resulted in increased ATP release from skin sections 15 minutes after barrier disruption, while acetone treatment led to ATP dependent increases in epidermal thickness and DNA synthesis in basal keratinocytes [48], [50], [51]. Moreover, a dry environment also induces epidermal thickening, while a humid environment has the opposite effect [51]. These changes in epidermal thickness may be due to alterations in keratinocyte ATP release through connexin hemichannels in response to humidity in the external environment. In addition, air-stimulated ATP release may participate in wound healing in response to tissue injuries such as cuts and abrasions, where keratinocyte contact with air is increased.

Importantly, air-stimulated ATP release may also underlie the formation of organotypic cultures. In these cultures, keratinocytes are brought to the air-liquid interface in order to create a thicker, more fully stratified epidermis compared to submerged cultures [22], [23]. The increased thickness of these organotypic cultures may result from air-mediated ATP release acting through P2Y receptors on basal keratinocytes, similar to the effects of ATP injection into the skin. The coordinated action of air-stimulated ATP release on both P2X and P2Y receptors may aid in the stratification of these cultures, which more closely models the *in vivo* epidermis than submerged cultures.

Air -stimulated ATP release through connexin hemichannels may contribute to dermatological pathologies that involve alterations in epidermal homeostasis and barrier formation. For example, hyper-proliferative diseases such as psoriasis may involve an increase in the air-stimulated release mechanism. Consistent with this, over-expression of connexin 26 in keratinocytes increased ATP release and caused morphological changes in the epidermis indicative of increased ATP signaling [52]. Connexin 26 over-expressing mice had a thicker epidermis, deficits in barrier formation and also displayed psoriasitic plaques.

### Potential Role for Air-stimulated ATP Release in Chronic Pain

In addition to a likely role in normal tactile sensation, there is accumulating evidence supporting the ability of keratinocyte signaling to influence pain sensation [53], [54]. Keratinocytes surround the terminals of nociceptive sensory neurons and release molecules that sensitize and activate these neurons (e.g. prostaglandin E_2_ and ATP), as well as those that inhibit their activity (e.g. β-endorphin) [6], [55]. We have previously shown that the peripheral anti-nociceptive effects of ET_B_ and CB_2_ receptor agonists likely involve the release of β-endorphin from keratinocytes, leading to reduced activity of μ opioid receptor-expressing nociceptive fibers [6], [56]. Normal tactile sensation, as well as acute and chronic pain, likely involve a balance between these keratinocyte excitatory and inhibitory neuromodulatory mechanisms.

Many of the signaling molecules that are released by keratinocytes help to regulate their own proliferation and differentiation, but also have the potential for contributing to pain sensation, especially when nociceptor sensitivity is enhanced under pathological conditions such as nerve injury. As described above, the keratinocyte cells that make up the epidermis release ATP to regulate epidermal homeostasis, a process that may be influenced by air-stimulated ATP release through connexin hemichannels. The cutaneous release of ATP, in particular, may make an important contribution to chronic pain [2].

Injection of ATP into the skin causes overt pain, most likely through the activation of P2X3 receptors expressed in small diameter neurites that innervate the superficial epidermis [1], [10], [57]. During inflammation, P2X3 receptors become sensitized in both animal models and human patients [57], [58], [59], [60]. Anti-sense knockdown of P2X3 in DRG results in reduced responses to ATP, as well as reduced thermal and mechanical hyperalgesia in inflammatory and neuropathic pain models [61], [62]. Furthermore, subcutaneous injection of P2X antagonists (e.g. the P2X3 specific inhibitor A-317491), are effective at reducing ongoing inflammatory and neuropathic pain, supporting a contribution of endogenous ATP release in the skin to chronic pain [13], [14], [15], [16]. P2Y receptors, especially P2Y1 and P2Y2, are also expressed by primary sensory endings and may participate in chronic pain states. For example, knockout of P2Y2 receptors prevents the development of CFA-induced thermal hyperalgesia, possibly through an interaction with TrpV1 [63].

Low levels of keratinocyte ATP released during epidermal stratification and in response to innocuous tactile stimulation may help regulate normal tactile sensation, but this release normally does not reach the level necessary to trigger a painful response [2]. In addition, purinergic antagonists often lack effects in acute mechanical and thermal nociceptive testing where an intense, escapable thermal or mechanical stimulus is applied to the skin (i.e. von Frey and hot plate tests) [14]. Thus, cutaneous ATP release does not appear to contribute to pain in the absence of tissue injury.

Under chronic pain conditions such as inflammation and nerve injury, however, nerve endings become sensitized and this normally innocuous level of epidermal ATP may now be sufficient to cause activation that reaches a nociceptive threshold [13]. Consequently, ATP released from keratinocytes in response to mild thermal and mechanical stimulation and during normal epidermal homeostasis may now contribute to tactile hypersensitivity and spontaneous pain.

Despite the likely importance of cutaneous ATP in chronic pain, the source of this ATP has remained unknown. Keratinocyte ATP release through connexin hemichannels represents a major potential source of this pro-algesic molecule. In fact, systemic administration of the connexin inhibitor carbenoxolone has been shown to reduce inflammatory pain in a CFA model, but this route of administration makes it unclear whether these effects could be due to reduced keratinocyte ATP release or through an exclusively central mechanism [64]. Future studies testing the cutaneous effects of connexin hemichannel blockers in animal models of chronic pain will help clarify a role for keratinocyte hemichannels in these conditions.

### Conclusion

In this study, we show that brief air exposure causes a robust ATP release from keratinocytes and provide strong evidence that this release occurs through connexin hemichannels. We propose that this mechanism is constantly at play during epidermal homeostasis and may also contribute to chronic pain through activation of purinergic receptors on sensitized epidermal nerve endings. Importantly, such an on-going release of ATP in the epidermis may contribute to spontaneous pain, which has traditionally been the most difficult aspect of human chronic pain to treat. It is also possible that keratinocyte ATP release in response to thermal and mechanical stimulation involves connexin hemichannels and contributes to tactile hypersensitivity under chronic pain conditions. Chronic pain and dermatological disease can often occur concomitantly and may involve common underlying pathologies in keratinocyte signaling. Further research into ATP release from keratinocytes will likely yield valuable insights into the sensory and structural functions of the epidermis and potentially lead to the identification of new targets for the treatment of chronic pain and dermatological disorders.
